# Diaqua­bis(2-chloro­benzoato-κ*O*)bis­(*N*,*N*-diethyl­nicotinamide-κ*N* ^1^)manganese(II)

**DOI:** 10.1107/S160053680901318X

**Published:** 2009-04-10

**Authors:** T. Hökelek, H. Dal, B. Tercan, F. E. Özbek, H. Necefoğlu

**Affiliations:** a Department of Physics, Hacettepe University, 06800 Beytepe, Ankara, Turkey; b Department of Chemistry, Faculty of Science, Anadolu University, 26470 Yenibağlar, Eskişehir, Turkey; c Department of Physics, Karabük University, 78050 Karabük, Turkey; d Department of Chemistry, Kafkas University, 63100 Kars, Turkey

## Abstract

In the monomeric title complex, [Mn(C_7_H_4_ClO_2_)_2_(C_10_H_14_N_2_O)_2_(H_2_O)_2_], the Mn^II^ atom is located on a crystallographic centre of inversion. The asymmetric unit contains one 2-chloro­benzoate (CB) ligand, one diethyl­nicotinamide (DENA) ligand and one coordinated water mol­ecule, all ligands being monodentate. The four O atoms in the equatorial plane around the Mn atom form a slightly distorted square-planar arrangement, while the slightly distorted octa­hedral coordination is completed by the two pyridine N atoms of the DENA ligands in the axial positions. The dihedral angle between the carboxyl group and the adjacent benzene ring is 77.9 (11)°, while the pyridine and benzene rings are oriented at a dihedral angle of 45.94 (5)°. In the crystal structure, inter­molecular O—H⋯O hydrogen bonds link the mol­ecules into infinite chains.

## Related literature

For general backgroud to transition metal complexes with biochemically active ligands, see: Antolini *et al.* (1982 ▶); Bigoli *et al.* (1972 ▶); Nadzhafov *et al.* (1981 ▶); Shnulin *et al.* (1981 ▶). For related structures, see: Hökelek *et al.* (1995 ▶, 1997 ▶, 2007 ▶, 2008 ▶); Hökelek & Necefoğlu (1996 ▶, 1997 ▶, 2007 ▶).
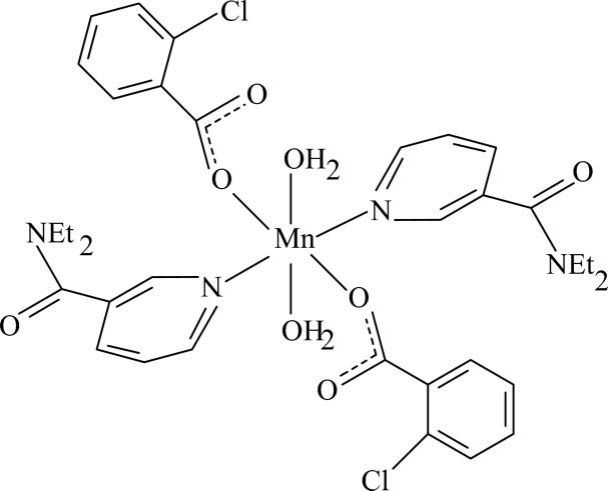


## Experimental

### 

#### Crystal data

[Mn(C_7_H_4_ClO_2_)_2_(C_10_H_14_N_2_O)_2_(H_2_O)_2_]
*M* *_r_* = 758.54
Monoclinic, 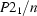

*a* = 13.2840 (2) Å
*b* = 10.2499 (3) Å
*c* = 15.0023 (4) Å
β = 114.988 (1)°
*V* = 1851.50 (8) Å^3^
*Z* = 2
Mo *K*α radiation
μ = 0.55 mm^−1^
*T* = 100 K
0.46 × 0.44 × 0.27 mm

#### Data collection

Bruker Kappa APEXII CCD area-detector diffractometer
Absorption correction: multi-scan (*SADABS*; Bruker, 2005 ▶) *T*_min_ = 0.778, *T*_max_ = 0.864
16951 measured reflections
4635 independent reflections
3984 reflections with *I* > 2σ(*I*)
*R*_int_ = 0.048

#### Refinement

*R*[*F* ^2^ > 2σ(*F* ^2^)] = 0.033
*wR*(*F* ^2^) = 0.091
*S* = 1.07
4635 reflections
233 parameters
3 restraints
H atoms treated by a mixture of independent and constrained refinement
Δρ_max_ = 1.17 e Å^−3^
Δρ_min_ = −0.38 e Å^−3^

### 

Data collection: *APEX2* (Bruker, 2007 ▶); cell refinement: *SAINT* (Bruker, 2007 ▶); data reduction: *SAINT*; program(s) used to solve structure: *SHELXS97* (Sheldrick, 2008 ▶); program(s) used to refine structure: *SHELXL97* (Sheldrick, 2008 ▶); molecular graphics: *ORTEP-3 for Windows* (Farrugia, 1997 ▶); software used to prepare material for publication: *WinGX* (Farrugia, 1999 ▶).

## Supplementary Material

Crystal structure: contains datablocks I, global. DOI: 10.1107/S160053680901318X/im2110sup1.cif

Structure factors: contains datablocks I. DOI: 10.1107/S160053680901318X/im2110Isup2.hkl

Additional supplementary materials:  crystallographic information; 3D view; checkCIF report

## Figures and Tables

**Table 1:** Hydrogen-bond geometry (Å, °)

*D*—H⋯*A*	*D*—H	H⋯*A*	*D*⋯*A*	*D*—H⋯*A*
O4—H41⋯O2^i^	0.90 (2)	1.83 (2)	2.666 (2)	154 (2)
O4—H42⋯O3^ii^	0.89 (2)	1.86 (2)	2.729 (1)	166 (2)

Symmetry codes: (i) 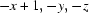; (ii) 
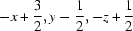
.

**Fig. 1.**
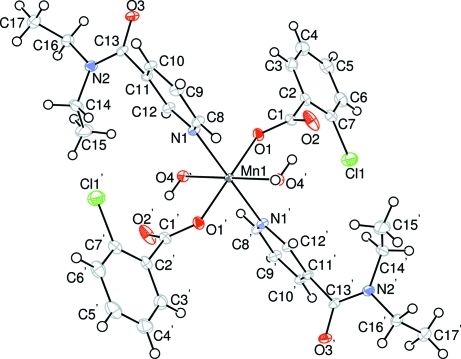
The molecular structure of the title molecule with the atom-numbering scheme. Displacement ellipsoids are drawn at the 50% probability level. Primed atoms are generated by the symmetry operator (1 - x, -y, -z).

## supplementary crystallographic information

### Comment

Transition metal complexes with biochemically active ligands frequently show interesting physical and/or chemical properties, as a result they may find applications in biological systems (Antolini *et al.*, 1982). The structural functions and coordination relationships of the arylcarboxylate ion in transition metal complexes of benzoic acid derivatives change depending on the nature and position of the substituent groups on the benzene ring, the nature of the additional ligand molecule or solvent, and the medium of the synthesis (Nadzhafov *et al.*, 1981; Shnulin *et al.*, 1981). The nicotinic acid derivative *N*,*N*-diethylnicotinamide (DENA) is an important respiratory stimulant (Bigoli *et al.*, 1972).

The structure determination of the title compound, (I), a manganese complex with two 2-chlorobenzoate (CB), two diethylnicotinamide (DENA) ligands and two water molecules, was undertaken in order to determine the properties of the ligands and also to compare the results obtained with those reported previously.

Compound (I) is a monomeric complex, with the Mn atom on a centre of symmetry. It contains two CB, two DENA ligands and two water molecules (Fig. 1). All ligands are monodentate. The four O atoms (O1, O4, and the symmetry-related atoms, O1', O4') in the equatorial plane around the Mn atom form a slightly distorted square-planar arrangement, while the slightly distorted octahedral coordination is completed by the two pyridine N atoms of the DENA ligands (N1, N1') in the axial positions (Fig. 1).

The near equality of the C1—O1 [1.260 (2) Å] and C1—O2 [1.238 (2) Å] bonds in the carboxylate group indicates a delocalized bonding arrangement, rather than localized single and double bonds, and may be compared with the corresponding distances: 1.256 (6) and 1.245 (6) Å in [Mn(DENA)_2_(C_7_H_4_ClO_2_)_2_(H_2_O)_2_] (Hökelek *et al.*, 2008), 1.265 (6) and 1.275 (6) Å in [Mn(C_9_H_10_NO_2_)_2_(H_2_O)_4_] × 2 H_2_O (Hökelek & Necefoğlu, 2007), 1.260 (4) and 1.252 (4) Å in [Zn(DENA)_2_(C_7_H_4_FO_2_)_2_(H_2_O)_2_] (Hökelek *et al.*, 2007), 1.259 (9) and 1.273 (9) Å in [Cu_2_(DENA)_2_(C_6_H_5_COO)_4_] (Hökelek *et al.*, 1995), 1.279 (4) and 1.246 (4) Å in [Zn_2_(DENA)_2_(C_7_H_5_O_3_)_4_] × 2 H_2_O (Hökelek & Necefoğlu, 1996), 1.251 (6) and 1.254 (7) Å in [Co(DENA)_2_(C_7_H_5_O_3_)_2_(H_2_O)_2_] (Hökelek & Necefoğlu, 1997) and 1.278 (3) and 1.246 (3) Å in [Cu(DENA)_2_(C_7_H_4_NO_4_)_2_(H_2_O)_2_] (Hökelek *et al.*, 1997). In the title compound, the average Mn—O bond length is 2.161 (1) Å and the Mn atom is displaced out of the least-squares plane of the carboxylate group (O1/C1/O2) by -0.544 (1) Å. The dihedral angle between the planar carboxylate group and the benzene ring A (C2—C7) is 77.9 (1)°, while that between rings A and B (N1/C8—C12) is 45.94 (5)°.

In the crystal structure, intermolecular O—H···O hydrogen bonds (Table 1) link the molecules into infinite chains.

### Experimental

The title compound was prepared by the reaction of MnSO_4_ × H_2_O (0.85 g, 5 mmol) in H_2_O (20 ml) and DENA (1.78 g, 10 mmol) in H_2_O (20 ml) with sodium 2-chlorobenzoate (1.785 g, 10 mmol) in H_2_O (50 ml). The mixture was filtered and set aside to crystallize at ambient temperature for 4 d, giving colorless single crystals.

### Refinement

H atoms of water molecule were located in difference Fourier maps and refined isotropically, with restrains of O4—H41 = 0.896 (12) and O4—H42 = 0.890 (15) Å and H41—O4—H42 = 105.1 (19)°. The remaining H atoms were positioned geometrically with C—H = 0.93, 0.97 and 0.96 Å, for aromatic, methylene and methyl H atoms and constrained to ride on their parent atoms, with *U*_iso_(H) = *xU*_eq_(C), where *x* = 1.5 for methyl H and *x* = 1.2 for all other H atoms.

### Crystal data

**Table:**

[Mn(C_7_H_4_ClO_2_)_2_(C_10_H_14_N_2_O)_2_(H_2_O)_2_]	*F*(000) = 790
*M**_r_* = 758.54	*D*_x_ = 1.361 Mg m^−^^3^
Monoclinic, *P*2_1_/*n*	Mo *K*α radiation, λ = 0.71073 Å
Hall symbol: -P 2yn	Cell parameters from 8796 reflections
*a* = 13.2840 (2) Å	θ = 2.5–28.4°
*b* = 10.2499 (3) Å	µ = 0.55 mm^−^^1^
*c* = 15.0023 (4) Å	*T* = 100 K
β = 114.988 (1)°	Block, colorless
*V* = 1851.50 (8) Å^3^	0.46 × 0.44 × 0.27 mm
*Z* = 2	

### Data collection

**Table:**

Bruker Kappa APEXII CCD area-detector diffractometer	4635 independent reflections
Radiation source: fine-focus sealed tube	3984 reflections with *I* > 2σ(*I*)
graphite	*R*_int_ = 0.048
φ and ω scans	θ_max_ = 28.4°, θ_min_ = 1.7°
Absorption correction: multi-scan (*SADABS*; Bruker, 2005)	*h* = −17→17
*T*_min_ = 0.778, *T*_max_ = 0.864	*k* = −13→13
16951 measured reflections	*l* = −18→20

### Refinement

**Table:**

Refinement on *F*^2^	Primary atom site location: structure-invariant direct methods
Least-squares matrix: full	Secondary atom site location: difference Fourier map
*R*[*F*^2^ > 2σ(*F*^2^)] = 0.033	Hydrogen site location: inferred from neighbouring sites
*wR*(*F*^2^) = 0.091	H atoms treated by a mixture of independent and constrained refinement
*S* = 1.07	*w* = 1/[σ^2^(*F*_o_^2^) + (0.0439*P*)^2^ + 0.6941*P*] where *P* = (*F*_o_^2^ + 2*F*_c_^2^)/3
4635 reflections	(Δ/σ)_max_ < 0.001
233 parameters	Δρ_max_ = 1.17 e Å^−^^3^
3 restraints	Δρ_min_ = −0.38 e Å^−^^3^

### Special details

**Table:**

Geometry. All e.s.d.'s (except the e.s.d. in the dihedral angle between two l.s. planes) are estimated using the full covariance matrix. The cell e.s.d.'s are taken into account individually in the estimation of e.s.d.'s in distances, angles and torsion angles; correlations between e.s.d.'s in cell parameters are only used when they are defined by crystal symmetry. An approximate (isotropic) treatment of cell e.s.d.'s is used for estimating e.s.d.'s involving l.s. planes.
Refinement. Refinement of *F*^2^ against ALL reflections. The weighted *R*-factor *wR* and goodness of fit *S* are based on *F*^2^, conventional *R*-factors *R* are based on *F*, with *F* set to zero for negative *F*^2^. The threshold expression of *F*^2^ > σ(*F*^2^) is used only for calculating *R*-factors(gt) *etc*. and is not relevant to the choice of reflections for refinement. *R*-factors based on *F*^2^ are statistically about twice as large as those based on *F*, and *R*- factors based on ALL data will be even larger.

### Fractional atomic coordinates and isotropic or equivalent isotropic displacement parameters (Å^2^)

**Table:**

	*x*	*y*	*z*	*U*_iso_*/*U*_eq_	
Mn1	0.5000	0.0000	0.0000	0.01025 (9)	
Cl1	0.18512 (4)	−0.13983 (4)	0.08159 (3)	0.02988 (11)	
O1	0.46966 (9)	−0.04216 (10)	0.12506 (7)	0.0170 (2)	
O2	0.35458 (12)	0.11652 (11)	0.12485 (10)	0.0316 (3)	
O3	0.93810 (8)	0.11259 (9)	0.39721 (7)	0.0163 (2)	
O4	0.61938 (8)	−0.16212 (10)	0.03910 (7)	0.0146 (2)	
H41	0.634 (2)	−0.173 (2)	−0.0135 (13)	0.053 (7)*	
H42	0.6079 (18)	−0.2422 (16)	0.0558 (15)	0.038 (6)*	
N1	0.64747 (10)	0.13022 (11)	0.09437 (8)	0.0142 (2)	
N2	0.98935 (11)	0.00083 (12)	0.29330 (9)	0.0175 (3)	
C1	0.40228 (12)	0.01065 (14)	0.15298 (10)	0.0158 (3)	
C2	0.38071 (12)	−0.06704 (14)	0.22885 (10)	0.0168 (3)	
C3	0.45941 (14)	−0.07053 (16)	0.32641 (11)	0.0230 (3)	
H3	0.5231	−0.0196	0.3459	0.028*	
C4	0.44378 (15)	−0.14936 (17)	0.39502 (12)	0.0274 (4)	
H4	0.4965	−0.1505	0.4600	0.033*	
C5	0.34943 (15)	−0.22608 (17)	0.36617 (13)	0.0279 (4)	
H5	0.3396	−0.2800	0.4117	0.033*	
C6	0.26970 (15)	−0.22309 (16)	0.26997 (12)	0.0247 (3)	
H6	0.2061	−0.2742	0.2506	0.030*	
C7	0.28603 (13)	−0.14272 (15)	0.20293 (11)	0.0198 (3)	
C8	0.64781 (12)	0.26020 (14)	0.08373 (10)	0.0161 (3)	
H8	0.5867	0.2994	0.0340	0.019*	
C9	0.73522 (12)	0.33862 (14)	0.14366 (10)	0.0167 (3)	
H9	0.7324	0.4284	0.1340	0.020*	
C10	0.82671 (12)	0.28205 (13)	0.21802 (10)	0.0150 (3)	
H10	0.8856	0.3329	0.2601	0.018*	
C11	0.82833 (11)	0.14699 (13)	0.22827 (10)	0.0136 (3)	
C12	0.73728 (12)	0.07548 (14)	0.16547 (10)	0.0146 (3)	
H12	0.7385	−0.0147	0.1730	0.017*	
C13	0.92349 (11)	0.08431 (13)	0.31200 (10)	0.0134 (3)	
C14	0.98096 (15)	−0.03017 (19)	0.19502 (12)	0.0299 (4)	
H14A	1.0537	−0.0218	0.1951	0.036*	
H14B	0.9320	0.0323	0.1482	0.036*	
C15	0.93702 (17)	−0.1674 (2)	0.16204 (15)	0.0459 (6)	
H15A	0.9385	−0.1857	0.0998	0.069*	
H15B	0.8621	−0.1737	0.1555	0.069*	
H15C	0.9828	−0.2294	0.2100	0.069*	
C16	1.08576 (12)	−0.05400 (15)	0.37696 (11)	0.0192 (3)	
H16A	1.1004	−0.1414	0.3606	0.023*	
H16B	1.0688	−0.0601	0.4337	0.023*	
C17	1.18844 (14)	0.02997 (17)	0.40244 (12)	0.0259 (4)	
H17A	1.2498	−0.0080	0.4570	0.039*	
H17B	1.1745	0.1161	0.4198	0.039*	
H17C	1.2059	0.0350	0.3466	0.039*	

### Atomic displacement parameters (Å^2^)

**Table:**

	*U*^11^	*U*^22^	*U*^33^	*U*^12^	*U*^13^	*U*^23^
Mn1	0.01013 (14)	0.01034 (14)	0.00924 (14)	−0.00008 (10)	0.00310 (11)	0.00017 (10)
Cl1	0.0296 (2)	0.0346 (2)	0.0235 (2)	−0.00607 (17)	0.00939 (16)	−0.00069 (15)
O1	0.0177 (5)	0.0201 (5)	0.0156 (5)	0.0057 (4)	0.0093 (4)	0.0049 (4)
O2	0.0518 (8)	0.0195 (6)	0.0413 (7)	0.0159 (5)	0.0369 (7)	0.0131 (5)
O3	0.0187 (5)	0.0141 (5)	0.0116 (5)	0.0021 (4)	0.0019 (4)	−0.0019 (4)
O4	0.0170 (5)	0.0107 (5)	0.0161 (5)	0.0004 (4)	0.0068 (4)	0.0007 (4)
N1	0.0140 (6)	0.0132 (6)	0.0127 (5)	−0.0008 (4)	0.0030 (5)	0.0004 (4)
N2	0.0168 (6)	0.0199 (6)	0.0127 (6)	0.0039 (5)	0.0031 (5)	−0.0002 (4)
C1	0.0193 (7)	0.0144 (7)	0.0163 (7)	−0.0006 (5)	0.0099 (6)	−0.0003 (5)
C2	0.0211 (7)	0.0145 (7)	0.0197 (7)	0.0043 (6)	0.0135 (6)	0.0028 (5)
C3	0.0220 (8)	0.0259 (8)	0.0227 (8)	0.0017 (6)	0.0111 (6)	0.0043 (6)
C4	0.0296 (9)	0.0337 (9)	0.0204 (8)	0.0064 (7)	0.0121 (7)	0.0087 (6)
C5	0.0381 (10)	0.0270 (9)	0.0276 (8)	0.0046 (7)	0.0226 (8)	0.0100 (7)
C6	0.0293 (9)	0.0221 (8)	0.0303 (8)	−0.0027 (7)	0.0198 (7)	0.0022 (6)
C7	0.0242 (8)	0.0198 (7)	0.0191 (7)	0.0017 (6)	0.0125 (6)	0.0006 (5)
C8	0.0147 (6)	0.0157 (7)	0.0142 (6)	0.0016 (5)	0.0025 (5)	0.0023 (5)
C9	0.0196 (7)	0.0111 (6)	0.0166 (7)	−0.0005 (5)	0.0050 (6)	0.0010 (5)
C10	0.0150 (6)	0.0141 (7)	0.0134 (6)	−0.0028 (5)	0.0035 (5)	−0.0020 (5)
C11	0.0136 (6)	0.0147 (6)	0.0102 (6)	0.0008 (5)	0.0029 (5)	0.0001 (5)
C12	0.0155 (6)	0.0120 (6)	0.0132 (6)	−0.0001 (5)	0.0031 (5)	0.0002 (5)
C13	0.0125 (6)	0.0099 (6)	0.0140 (6)	−0.0027 (5)	0.0018 (5)	−0.0001 (5)
C14	0.0262 (9)	0.0466 (11)	0.0154 (7)	0.0147 (8)	0.0072 (6)	−0.0029 (7)
C15	0.0322 (10)	0.0573 (13)	0.0357 (10)	0.0100 (9)	0.0022 (8)	−0.0289 (9)
C16	0.0181 (7)	0.0180 (7)	0.0174 (7)	0.0069 (6)	0.0037 (6)	0.0022 (5)
C17	0.0189 (8)	0.0314 (9)	0.0235 (8)	0.0012 (7)	0.0051 (6)	−0.0036 (6)

### Geometric parameters (Å, °)

**Table:**

Mn1—O1	2.1233 (10)	C6—H6	0.9300
Mn1—O1^i^	2.1233 (10)	C7—C2	1.386 (2)
Mn1—O4^i^	2.1987 (10)	C7—C6	1.386 (2)
Mn1—O4	2.1987 (10)	C8—C9	1.3859 (19)
Mn1—N1^i^	2.2980 (12)	C8—H8	0.9300
Mn1—N1	2.2980 (12)	C9—C10	1.3828 (19)
Cl1—C7	1.7464 (16)	C9—H9	0.9300
O1—C1	1.2595 (18)	C10—H10	0.9300
O2—C1	1.2375 (18)	C11—C10	1.3920 (19)
O3—C13	1.2435 (17)	C11—C12	1.3875 (19)
O4—H41	0.896 (12)	C12—H12	0.9300
O4—H42	0.890 (15)	C13—C11	1.4991 (18)
N1—C8	1.3420 (18)	C14—C15	1.523 (3)
N1—C12	1.3419 (17)	C14—H14A	0.9700
N2—C13	1.3355 (19)	C14—H14B	0.9700
N2—C14	1.466 (2)	C15—H15A	0.9600
N2—C16	1.4742 (18)	C15—H15B	0.9600
C2—C1	1.512 (2)	C15—H15C	0.9600
C2—C3	1.394 (2)	C16—C17	1.519 (2)
C3—C4	1.392 (2)	C16—H16A	0.9700
C3—H3	0.9300	C16—H16B	0.9700
C4—C5	1.385 (3)	C17—H17A	0.9600
C4—H4	0.9300	C17—H17B	0.9600
C5—H5	0.9300	C17—H17C	0.9600
C6—C5	1.384 (2)		
			
O1—Mn1—O1^i^	180.00 (8)	C6—C7—Cl1	118.68 (13)
O1—Mn1—O4^i^	90.28 (4)	C6—C7—C2	122.05 (15)
O1^i^—Mn1—O4^i^	89.72 (4)	N1—C8—C9	122.86 (13)
O1—Mn1—O4	89.72 (4)	N1—C8—H8	118.6
O1^i^—Mn1—O4	90.28 (4)	C9—C8—H8	118.6
O1—Mn1—N1^i^	89.75 (4)	C8—C9—H9	120.3
O1^i^—Mn1—N1^i^	90.25 (4)	C10—C9—C8	119.35 (13)
O1—Mn1—N1	90.25 (4)	C10—C9—H9	120.3
O1^i^—Mn1—N1	89.75 (4)	C9—C10—C11	118.26 (13)
O4^i^—Mn1—O4	180.00 (6)	C9—C10—H10	120.9
O4^i^—Mn1—N1^i^	86.76 (4)	C11—C10—H10	120.9
O4—Mn1—N1^i^	93.24 (4)	C12—C11—C10	118.82 (13)
O4^i^—Mn1—N1	93.24 (4)	C12—C11—C13	121.82 (12)
O4—Mn1—N1	86.76 (4)	C10—C11—C13	119.15 (12)
N1^i^—Mn1—N1	180.00 (10)	N1—C12—C11	123.10 (13)
Mn1—O4—H41	105.2 (15)	N1—C12—H12	118.5
Mn1—O4—H42	125.6 (14)	C11—C12—H12	118.5
H41—O4—H42	105.1 (19)	O3—C13—N2	122.25 (12)
C1—O1—Mn1	129.29 (9)	O3—C13—C11	118.14 (13)
C8—N1—Mn1	123.24 (9)	N2—C13—C11	119.60 (12)
C12—N1—Mn1	119.15 (9)	N2—C14—C15	112.51 (16)
C12—N1—C8	117.59 (12)	N2—C14—H14A	109.1
C13—N2—C14	124.89 (12)	N2—C14—H14B	109.1
C13—N2—C16	118.42 (12)	C15—C14—H14A	109.1
C14—N2—C16	116.32 (13)	C15—C14—H14B	109.1
O1—C1—C2	114.28 (12)	H14A—C14—H14B	107.8
O2—C1—O1	126.65 (14)	C14—C15—H15A	109.5
O2—C1—C2	119.07 (13)	C14—C15—H15B	109.5
C3—C2—C1	120.47 (14)	C14—C15—H15C	109.5
C7—C2—C1	121.43 (13)	H15A—C15—H15B	109.5
C7—C2—C3	117.99 (14)	H15A—C15—H15C	109.5
C2—C3—H3	119.6	H15B—C15—H15C	109.5
C4—C3—C2	120.76 (16)	N2—C16—C17	111.28 (13)
C4—C3—H3	119.6	N2—C16—H16A	109.4
C3—C4—H4	120.1	N2—C16—H16B	109.4
C5—C4—C3	119.78 (15)	C17—C16—H16A	109.4
C5—C4—H4	120.1	C17—C16—H16B	109.4
C4—C5—H5	119.8	H16A—C16—H16B	108.0
C6—C5—C4	120.42 (15)	C16—C17—H17A	109.5
C6—C5—H5	119.8	C16—C17—H17B	109.5
C5—C6—C7	118.98 (16)	C16—C17—H17C	109.5
C5—C6—H6	120.5	H17A—C17—H17B	109.5
C7—C6—H6	120.5	H17A—C17—H17C	109.5
C2—C7—Cl1	119.26 (11)	H17B—C17—H17C	109.5
			
O1—Mn1—N1—C8	−112.21 (12)	C3—C2—C1—O1	75.74 (18)
O1^i^—Mn1—N1—C8	67.79 (12)	C3—C2—C1—O2	−104.05 (18)
O1—Mn1—N1—C12	66.18 (11)	C7—C2—C1—O1	−100.36 (17)
O1^i^—Mn1—N1—C12	−113.82 (11)	C7—C2—C1—O2	79.85 (19)
O4^i^—Mn1—O1—C1	2.94 (12)	C1—C2—C3—C4	−175.23 (15)
O4—Mn1—O1—C1	−177.06 (12)	C7—C2—C3—C4	1.0 (2)
O4^i^—Mn1—N1—C8	−21.92 (12)	C2—C3—C4—C5	0.5 (3)
O4—Mn1—N1—C8	158.08 (12)	C3—C4—C5—C6	−1.1 (3)
O4^i^—Mn1—N1—C12	156.47 (11)	C7—C6—C5—C4	0.3 (3)
O4—Mn1—N1—C12	−23.53 (11)	C6—C7—C2—C1	174.34 (14)
N1^i^—Mn1—O1—C1	−83.82 (12)	C6—C7—C2—C3	−1.8 (2)
N1—Mn1—O1—C1	96.18 (12)	Cl1—C7—C2—C1	−4.3 (2)
Mn1—O1—C1—O2	−16.0 (2)	Cl1—C7—C2—C3	179.50 (12)
Mn1—O1—C1—C2	164.20 (9)	C2—C7—C6—C5	1.2 (2)
Mn1—N1—C8—C9	177.23 (11)	Cl1—C7—C6—C5	179.86 (13)
C12—N1—C8—C9	−1.2 (2)	N1—C8—C9—C10	0.0 (2)
Mn1—N1—C12—C11	−177.57 (11)	C8—C9—C10—C11	1.5 (2)
C8—N1—C12—C11	0.9 (2)	C12—C11—C10—C9	−1.7 (2)
C14—N2—C13—O3	−175.90 (15)	C13—C11—C10—C9	−176.41 (13)
C16—N2—C13—O3	−3.2 (2)	C10—C11—C12—N1	0.5 (2)
C14—N2—C13—C11	4.1 (2)	C13—C11—C12—N1	175.09 (13)
C16—N2—C13—C11	176.82 (12)	O3—C13—C11—C10	62.17 (19)
C13—N2—C14—C15	−108.92 (18)	O3—C13—C11—C12	−112.38 (16)
C16—N2—C14—C15	78.20 (18)	N2—C13—C11—C10	−117.81 (15)
C13—N2—C16—C17	−90.68 (17)	N2—C13—C11—C12	67.64 (19)
C14—N2—C16—C17	82.69 (17)		

Symmetry codes: (i) −*x*+1, −*y*, −*z*.

### Hydrogen-bond geometry (Å, °)

**Table:**

*D*—H···*A*	*D*—H	H···*A*	*D*···*A*	*D*—H···*A*
O4—H41···O2^i^	0.90 (2)	1.83 (2)	2.666 (2)	154 (2)
O4—H42···O3^ii^	0.89 (2)	1.86 (2)	2.729 (1)	166 (2)

Symmetry codes: (i) −*x*+1, −*y*, −*z*; (ii) −*x*+3/2, *y*−1/2, −*z*+1/2.
